# Substance Use Disorder Status Moderates the Association between Personality Traits and Problematic Mobile Phone/Internet Use

**DOI:** 10.3390/jcm10050919

**Published:** 2021-02-26

**Authors:** Marta Demkow-Jania, Maciej Kopera, Elisa M. Trucco, Paweł Kobyliński, Anna Klimkiewicz, Małgorzata Abramowska, Anna Mach, Andrzej Jakubczyk

**Affiliations:** 1 Department of Psychiatry, Medical University of Warsaw, 00-665 Warsaw, Poland; demkowmarta@gmail.com (M.D.-J.); maciej.kopera@wum.edu.pl (M.K.); aklimkiewicz@wum.edu.pl (A.K.); malgorzata.abramowska@wum.edu.pl (M.A.); andrzej.jakubczyk@wum.edu.pl (A.J.); 2 Department of Psychology, Center for Children and Families, Florida International University, Miami, FL 33199, USA; etrucco@fiu.edu; 3 Department of Psychiatry, Addiction Center, University of Michigan, Ann Arbor, MI 48109, USA; 4 National Information Processing Institute, Laboratory of Interactive Technologies, 00-608 Warsaw, Poland; pawel.kobylinski@opi.org.pl

**Keywords:** problematic mobile phone use, substance use disorder, personality, neuroticism, openness to new experience

## Abstract

Background: Associations between personality traits and problematic smartphone use (PSU) among individuals with substance use disorder (SUD) have not been widely investigated. The current study aims to assess whether SUD status moderates the association between personality traits and PSU. Methods: The study group included 151 individuals with SUD and a normative sample (NS) comprised of 554 non-SUD students. The following self-report questionnaires were used: the Mobile Phone Problem Use Scale (MPPUS-10) to assess problematic smartphone use (PSU), the Internet Addiction Test (IAT) to assess intensity of internet use, and the NEO Five-Factor Inventory (NEO-FFI) to assess Personality traits. Results: SUD status moderated the association between neuroticism and openness to new experiences on PSU. That is, greater neuroticism and openness were significantly associated with more excessive PSU among the NS. In the SUD group, greater openness was a significant protective factor against PSU. Moderation results were similar when using the IAT (which was significantly correlated with MPPUS) as an outcome. Conclusions: The presence of SUD may influence how personality traits are associated with problematic mobile phone/internet use. Given that this is among one of the first studies examining this topic, findings should be replicated with additional studies.

## 1. Introduction

In January 2019, 4388 billion people reported using the internet, while 5112 billion owned a mobile phone, which is indicative of today’s digitization [1]. Almost all children (96.6%) have regular contact with mobile devices and approximately one in ten have already used the Internet before age 1 [2,3]. On average, most internet users spend 6 h and 42 min online each day, which equates to a total of more than 100 days online each year [1]. Early usage of technological devices and the corresponding prolonged exposure to screen time is reflective of their deleterious impact on public health. Moreover, technological addictions, which include problematic smartphone use (PSU) and problematic internet use (PIU), have begun to gain importance nowadays. Symptoms of problematic use of modern technologies include both physical, as well as psychological/psychiatric, manifestations. Muscle pain, ocular afflictions resulting from Computer Vision Syndrome (e.g., fatigue, dryness, blurry vision, irritation, or ocular redness), and pain and weakness in the thumbs and wrists leading to an increased number of cases of de Quervain’s tenosynovitis, are examples of physical signs [4,5]. Insomnia and sleep disturbances, anxiety and loneliness when unable to send a message or receive an immediate response and auditory and tactile illusions reflect common psychological/psychiatric symptoms of PSU/PIU [6,7,8].

Currently, the Diagnostic and Statistical Manual of Mental Disorders Fifth Edition (DSM-5) and the International Classification of Diseases (ICD-10) do not recognize PSU as a separate diagnosis. Yet, given the significant amount of time spent on smartphones/internet, as well as the possible severe consequences of PSU/PIU, it seems increasingly necessary to acknowledge this problem and develop therapeutic programs that could effectively inform tailored interventions based on the personality profile of the patient [9,10,11]. However, current work on associations between features of personality and symptoms of PSU is scarce and inconsistent. Among the trait-related personality conceptualizations, the most well-known dimensional personality model is the Big Five, which encompasses the following personality domains: extraversion; neuroticism; openness to new experiences; agreeableness; and conscientiousness [12].

According to Takao [13], PSU is related to higher extraversion, higher neuroticism, and lower openness to new experiences, while it is not associated with agreeableness or conscientiousness. In a study conducted among college students in 2005, Bianchi and Phillips found that younger age, high extraversion and low self-esteem, but not neuroticism, were related to PSU [14]. In 2019, Lachmann and colleagues found that similar personality traits, were associated with problematic smartphone and internet use [15]. PSU and PIU were associated with high neuroticism and low conscientiousness, as well as low agreeableness. Yet, symptoms of PIU (but not PSU) were negatively correlated with extraversion. Lachmann and colleagues speculate that individuals high on extraversion may be more likely to connect with their immediate environment, which leads to decreased computer use. Yet, this was not the case for smartphone use as extraverts tend to utilize their smartphones as an extension of their social self [15]. At the same time, symptoms of PSU (but not PIU) were negatively correlated with openness to new experiences. Results of a meta-analytic review published in 2020 demonstrated that high neuroticism, high extraversion, low agreeableness and low conscientiousness were associated with PSU [16].

Still, the international scientific community disagrees on whether behavioral and substance use addictions should be treated similarly. Apart from the common diagnostic criteria (e.g., impaired control, ongoing involvement despite negative consequences or symptoms of distress, which appear when a specific substance or behavior is unavailable), both disorders were also shown to have a similar neurobiological underpinning within the mesolimbic pathway. In both cases, similar activation in the reward circuit and associated regions, including the amygdala, hippocampus and frontal cortex, is induced by natural rewards (behaviors) or substances. The results of genetic testing and analysis of family history, though limited with respect to behavioral addictions, provide further evidence of similarities. For instance, pathological gambling and substance use disorder (SUD) are both highly heritable with a similar degree of heritability across biological sex. In both cases, small, additive effects across multiple neurotransmitter genes and associations with polymorphisms of dopamine receptor genes, which impact a range of brain functions, increase risk [17,18]. Yet, some researchers that are against treating substance use and behavioral addictions as similar phenomena indicate that the presence of physical withdrawal symptoms is unique to SUD and therefore substance use should be considered separately from problematic use of technology [9,18,19]. Moreover, the mechanisms underlying misuse of different substances likely vary. This also applies to personality traits. For instance, an Austrian study examining a sample of men (33 polydrug users, 30 alcohol users), found significant differences in personality traits across groups based on substance of choice. While a high level of neuroticism and a low level of openness to new experiences were found in both groups compared to a normative sample, a low level of conscientiousness and a low level of agreeableness was only found when comparing polydrug users to a normative sample [20].

Despite the aforementioned inconsistencies, similarities in symptomatology between PSU/PIU and SUD highlight the utility in exploring possible connections between problematic substance use and PSU/PIU. Surprisingly, there are only a few studies addressing this issue. A notable exception is a Swiss study conducted on 5096 men, which found that the prevalence of risky single-occasion drinking was positively associated with PSU, while both the frequent use of cannabis and daily smoking were negatively associated with PSU [21].

Personality traits play an important role in the development, duration and prognosis of SUD [22]. Therefore, investigations relating to personality features may contribute to treatment; specifically, implementation of personalized clinical interventions. Current knowledge on PSU is based mainly on data collected from college students, which may not accurately reflect the personality profile of all problematic smartphone users. The association between the use of psychoactive substances and technological addictions is often included as an addendum to studies that aim to assess more general factors relating to a healthy lifestyle. There are, however, a few reports that specifically investigate the co-occurrence of PSU and the use of psychoactive substances. A recent Polish study found that individuals with SUD did not differ from healthy controls in terms of mobile phone intensity [23]. However, prior work has not investigated differences across individuals with SUD and healthy controls with respect to associations between personality traits and problematic smartphone use to our knowledge. Current work indicates that different addictions (e.g., substance use, behavioral) may stem from distinct processes involved in personality development [24] and that substance use may affect risk of problematic mobile phone use [21]. Yet, it is unclear whether the association between personality profile and mobile phone use may be impacted by substance use. This is plausible given that substance use and behavioral addictions may arise from similar underlying drives (e.g., stimulation or regulation of negative affect). Therefore, the aim of the current study was to investigate the association between Big Five personality traits and problematic mobile phone use, and to assess whether SUD status (i.e., individuals with SUD vs. healthy controls) moderates this association. Given that this is one of the first studies examining interactions between personality traits and SUD status on problematic mobile phone use, a priori hypotheses were not generated.

## 2. Material and Methods

### 2.1. Procedures

Study procedures were conducted according to the ethical principles of the Declaration of Helsinki in 1964 amended by the 64th WMA General Assembly, Fortaleza, Brazil, October 2013 and received approval from the Bioethics Committee at the Medical University of Warsaw. All participants were volunteers recruited from either the Substance Abuse Treatment Center at the Nowowiejski Hospital during the course of their hospitalization or from the Medical University of Warsaw Poland during an introductory class in addiction medicine or psychiatry as part of their 1st and 4th year of medical school. Financial compensation was not provided for participation. Volunteers were asked to complete a series of surveys between January and June 2017. The average time to complete the questionnaire was 20 min for the normative sample (NS; students) and 30 min for the substance use disorder (SUD) group. All respondents were informed about the aims of the study. All participants needed to be at least 18 years or older to participate. Participants completed a consent process prior to study enrollment. Exclusion criteria included: a lack of informed consent, age 17 years or younger and an inability to complete the survey or to understand the purpose of the research.

### 2.2. Participants

The NS group consisted of 554 students (39.7% males), 18 to 25 years of age, from the Medical University of Warsaw Poland who did not meet criteria for SUD with the exception of nicotine and caffeine use (not assessed). Medical students were chosen as healthy controls because they commonly use smartphones as a communication and learning tool (e.g., for listening to or reading medical information, for downloading scientific materials).

The sample of individuals with SUD consisted of 151 Polish individuals (70.2% males), 19 to 41 years of age, who were admitted to the Substance Abuse Treatment Center for Methadone Maintenance Therapy. A diagnosis of SUD was based on the ICD-10 diagnostic criteria [25] and was established by medical specialists during their admission to the unit and later confirmed by a member of the research team. Questionnaires were administered to participants by clinicians during their hospitalization. All patients included in the current study were poly-drug users whose drug of choice was opioids. Still, these participants reported occasional use of amphetamine, cocaine, cannabinoids, and new psychoactive substances (mephedrone, synthetic cannabinoids, cathinones, etc.).

### 2.3. Measures

The questionnaire format was self-report and included questions regarding sociodemographic variables, personality traits and structured scales to assess drug use and online activities using their mobile phone. Specifically, questionnaires included: The Mobile Phone Problem Use Scale (MPPUS-10) to assess problematic smartphone use, the Internet Addiction Test (IAT) to assess intensity of internet use, the NEO Five-Factor Inventory (NEO-FFI) to assess personality traits, and the Drug Use Disorder Identification Test (DUDIT) to assess problematic drug use.

#### 2.3.1. Problematic Mobile Phone Use

The Polish version of the MPPUS-10 [26] was used to assess problematic mobile phone use. The MPPUS-10 is a 10-item measure with a 10-point Likert scale ranging from 1 (“not true at all”) to 10 (“extremely true”). It contains questions concerning loss of control, withdrawal, negative life consequences, craving and peer dependence. Sample items include: “I find myself engaged on a mobile phone for longer periods of time than intended” (loss of control); “I have used my mobile phone to make myself feel better when I was feeling down” (craving); “when out of range for some time, I become preoccupied with the thought of missing a call” (withdrawal) [26,27]. Internal consistency was good within this sample (Cronbach’s α = 0.78). The MPPUS-10 score is intended to reflect mobile phone use intensity on a continuum and is not considered a diagnostic tool. Therefore, a threshold for problematic use was not adopted.

#### 2.3.2. Internet Addiction

Internet addiction was assessed using the IAT [28]. This is a 20-item measure with a 5-point scale ranging from 1 (“very rarely”) to 5 (“very frequently”). It includes questions regarding the frequency of internet use, its impact on mood, relations with the environment, symptoms resulting from lack of internet access, and control over time spent on the Internet. The validated Polish version of the IAT was used [28,29]. Internal consistency was good within this sample (Cronbach’s α = 0.91). In this study, the IAT was used to determine whether findings are consistent across both intensity of internet use, as well as smartphone use.

#### 2.3.3. Personality Traits

Personality traits were assessed using the NEO-FFI [12], a 60-item short form of the 240-item NEO-PI-R. More specifically, the Polish S-version (self-report) of the NEO-FFI validated by Zawadzki et al. was administered [30]. This questionnaire contains a 5-point scale ranging from 1 (“definitely false”) to 5 (“definitely true”). Subscales of the measure consist of 12 questions each, reflecting each of the Big Five personality traits: neuroticism (Cronbach’s alpha α = 0.88), extraversion (Cronbach’s alpha α = 0.77), openness to experiences (Cronbach’s alpha α = 0.66), agreeableness (Cronbach’s alpha α = 0.70), and conscientiousness (Cronbach’s alpha α = 0.89).

#### 2.3.4. Substance Use Disorder

The Polish version of the 11-item DUDIT [31] was used to confirm SUD in the clinical sample and to exclude individuals with SUD symptoms in the NS. The first nine questions are scored on a 5-point scale ranging from 0 to 4, representing: 0 (“Never”), 1 (“Once a month or less often”), 2 (“2–4 times a month”), 3 (“2–3 times a week”), 4 (“4 times a week or more often”). For example, “How often do you use drugs other than alcohol?” The last two items are scored on a 3-point scale with values 0, 2, and 4, representing: 0 (“No”), 2 (“Yes, but not over the past year”), and 4 (“Yes, over the last year”). For example, “Have you or anyone else been hurt (mentally or physically) because you used drugs?” Higher scores indicate greater drug problem severity (score range 0–44). Internal consistency was good within this sample (Cronbach’s α = 0.92).

### 2.4. Data Analysis

**Figure 1:**
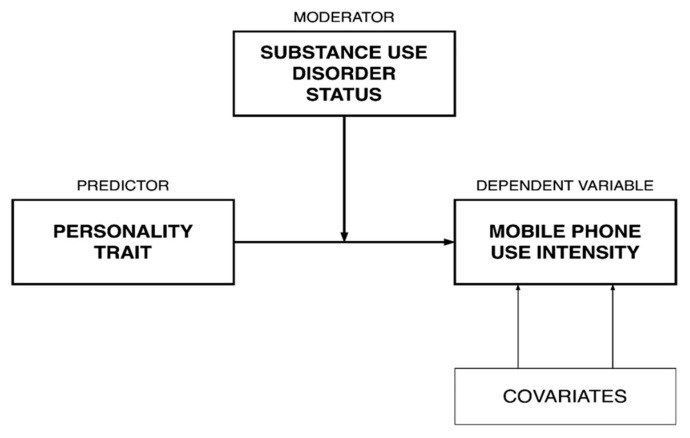
Conceptual diagram for moderation models.

First, the normative sample was compared to individuals with SUD in terms of basic demographic factors (e.g., age, biological sex), as well as clinical characteristics (i.e., intensity of mobile phone use [MPPUS], intensity of internet use [IAT], personality traits [NEO-FFI], and drug problem severity [DUDIT]). All continuous data were checked for normality using the Shapiro-Wilk test. For parametric variables, means and standard deviations (mean ± SD) are presented. Comparisons between groups were conducted using a one-way analysis of variance (ANOVA). For non-parametric variables, medians and quartiles (25; 75) are presented. Comparisons between groups were conducted using Mann-Whitney U tests. Subsequently, five separate models were tested with SUD status as a potential moderator of the association between each of the five NEO-FFI subscales and smartphone use intensity. The PROCESS macro for moderation analysis with bootstrapping (5000 resamples with replacement) in SPSS was used. Demographic characteristics that differed across the groups were included as potential covariates. Figure 1 illustrates the conceptual diagram for the models. A Bonferroni correction was applied to account for multiple testing. Next, simple slope analyses reflecting a “pick-a-point” approach were conducted to probe significant interactions [32]. Non-standardized coefficients are reported throughout the paper.

Personality reflects the following five traits—openness to experiences, conscientiousness, extraversion, agreeableness, neuroticism. Substance use disorder status reflects those meeting criteria for a substance use disorder versus healthy controls. Mobile phone use intensity is assessed using the Mobile Phone Problem Use Scale (MPPUS-10). Covariates include: biological sex and age.

## 3. Results

Group comparisons revealed that the SUD group was significantly older and consisted of significantly more men than the healthy control group (see Table 1 for details). There were no significant differences between groups in terms of mobile phone (MPPUS-10) and internet (IAT) use. As expected, the SUD group scored significantly higher on the DUDIT. The comparison of personality traits showed that individuals with SUD were significantly more neurotic, less open, less conscientious, and less agreeable in comparison to the NS.

### 3.1. Moderation Models

Support for SUD status as a moderator in the association between neuroticism and openness to experiences on intensity of mobile phone use was found. These two models are described in more detail below.

#### 3.1.1. Model A: Openness to Experiences, SUD Status, and Smartphone Use Intensity

**Figure 2:**
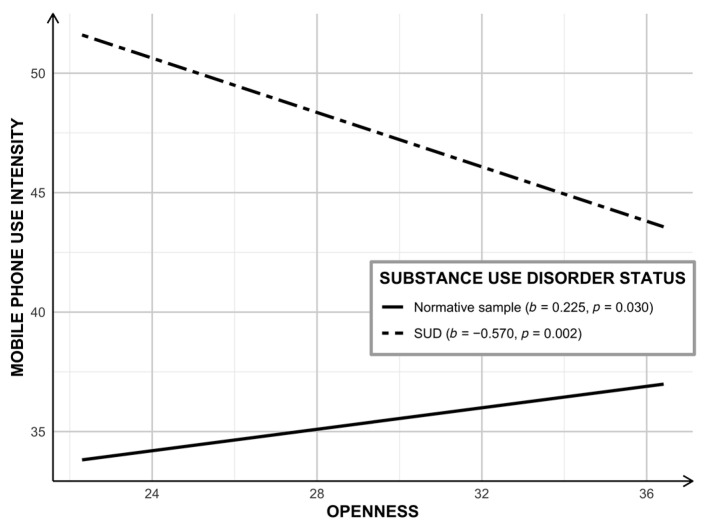
Openness to new experiences on smartphone use intensity by substance use disorder status. The simple slope of openness to new experiences on mobile phone use intensity was significant, yet positive, for those in the normative sample (solid line). The simple slope of openness to new experiences on mobile phone use intensity was significant, yet negative, for those in the SUD group (dashed lined). SUD—Substance Use Disorder.

In the first model, the role of SUD status as a moderator of the association between o*penness to experiences* and smartphone use intensity was tested with age and biological sex as covariates. The model explained 7% of the variance in smartphone use intensity (*R*^2^ = 0.074; *F*[5, 696] = 11.079; *p* < 0.001). A significant interaction was found between openness and SUD status (*b* = −0.400; 95% CI = [−0.601, −0.193]; *p* < 0.001; Δ*R*^2^ = 0.019). When probing the interaction (Figure 2), findings indicate that the simple slopes for the regression of smartphone use intensity on openness to experiences were statistically significant for both groups. Yet, the slope was positive among the NS (*b* = 0.225; 95% CI = [0.022, 0.427]; *p* = 0.030) and negative among individuals with SUD (*b* = −0.570; 95% CI = [−0.923, −0.215]; *p* = 0.002).

#### 3.1.2. Model B: Neuroticism, SUD Status, and Smartphone Use Intensity

**Figure 3:**
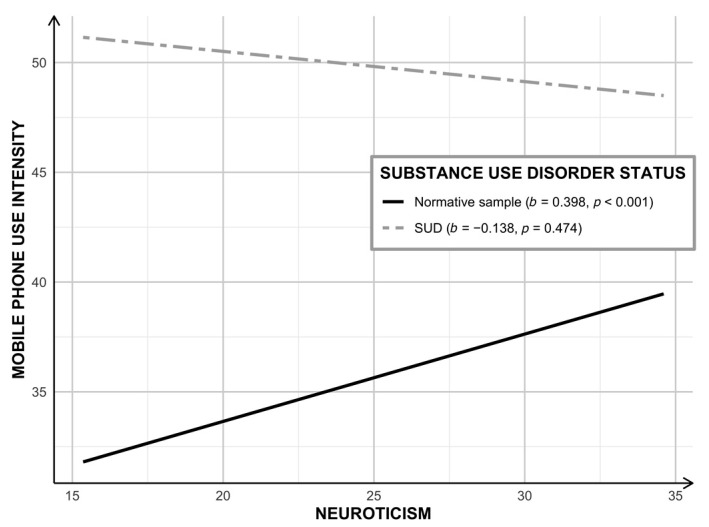
Neuroticism on smartphone use disorder intensity by substance use disorder status. The simple slope of neuroticism on mobile phone use intensity was significant, yet positive, for those in the normative sample (solid line). The simple slope of neuroticism on mobile phone use intensity was not significant for those in the SUD group (dashed line). SUD—Substance Use Disorder.

In the second model, the role of SUD status as a moderator of the association between neuroticism and smartphone use intensity was tested with age and biological sex as covariates. The model explained 10% of the variance in smartphone use intensity (*R*^2^ = 0.098; *F*[5, 696] = 15.060; *p* < 0.001). A significant interaction was found between neuroticism and SUD status (*b* = −0.268; 95% CI = [−0.469, −0.067]; *p* = 0.009; Δ*R*^2^ = 0.009). When probing the interaction (Figure 3), findings indicate that the simple slope for the regression of smartphone use intensity on neuroticism was statistically significant for the NS, but not for individuals with SUD. Within the normative sample, neuroticism was positively associated with smartphone use intensity (*b* = 0.398; 95% CI = [0.262, 0.534]; *p* < 0.001).

**Figure 4:**
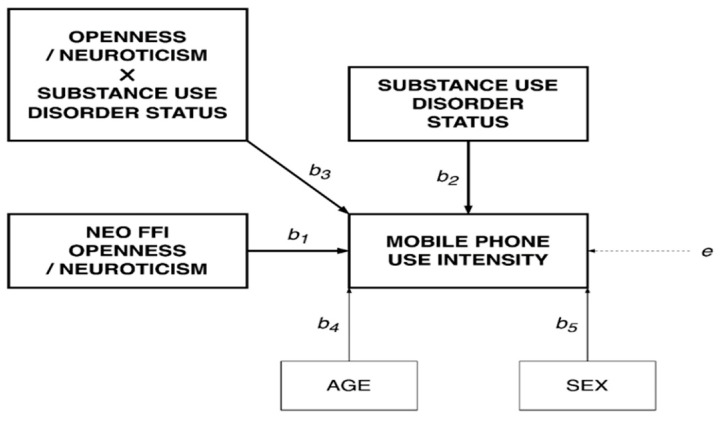
Effects of NEO Five-Factor Inventory (NEO-FFI) openness to new experience and neuroticism and substance use disorder status (i.e., individuals with a SUD vs. healthy controls) on mobile phone use intensity. Age and biological sex were included as covariates.

Figure 4. illustrates the statistical diagram for the tested moderated models. Table 2 contains detailed information about the resulting non-standardized coefficients.

There was no evidence for significant interactions across the other personality domains when the Bonferroni correction for multiple testing was applied.

### 3.2. Additional Analyses

Given the strong correlation between the IAT and the MMPUS (* *r* = 0.50; *p* < 0.0005 for both groups), additional models were estimated to determine whether findings generalize to internet use. Findings from moderation models with IAT scores as the outcome were largely consistent with those found with MPPUS as the outcome (i.e., significant interaction effects for neuroticism and openness by SUD status).

## 4. Discussion

The main aim of the present study was to investigate the association between personality traits and problematic mobile phone use in a normative sample (medical students) and among individuals with a SUD (mostly reporting opioids as a drug of choice, but commonly using additional substances). Our results indicated that there were no significant differences in level of PSU between students and individuals with SUD. In terms of comparisons of personality traits across the different samples, individuals with SUD were significantly more neurotic, less open to new experiences, less conscientious, and less agreeable than the NS. Moreover, in the case of neuroticism and openness, SUD status moderated the association between personality and PSU severity. Namely, greater neuroticism and openness to new experiences were significantly associated with more excessive PSU in the normative group. In the SUD group, greater openness to new experiences was a protective factor against PSU and there was no association between neuroticism and PSU. Finally, as expected, internet and smartphone use were significantly correlated in both groups. Moreover, SUD status was found to significantly moderate the association between neuroticism and openness to new experiences on internet use, consistent with the smartphone use models. To the best of our knowledge, this is the first study to investigate associations between personality traits and problematic mobile phone/internet use among individuals with SUD.

### 4.1. Neuroticism

Higher neuroticism in individuals with SUD when compared to healthy individuals corresponds with prior work [33]. However, statistical analyses within groups showed a positive correlation between neuroticism and PSU among the normative sample. This result is consistent with prior work [15,34]. Interestingly, an opposite association was found between neuroticism and PSU among the SUD group.

Neuroticism is represented by characteristics such as vulnerability, anxiety and a tendency towards greater depression [12]. This trait is also related to low self-esteem and a need for social approval. Consequently, it may be that students characterized by high levels of neuroticism are able to cope with negative emotional states effectively through the use of their smartphone [35].

The SUD group considered in the current study consisted of poly-drug users, who met criteria for an opioid use disorder. According to the literature, individuals with SUD that score high on neuroticism, are more likely to use sedatives and opiates in order to alleviate symptoms of depression and anxiety [36,37]. Therefore, it can be speculated that individuals with an opioid use disorder may prefer to use substances over the use of their smartphone/internet to cope with negative emotions. This is in line with findings that the level of negative emotions (higher neuroticism) was significantly higher among individuals with SUD compared to the NS. Therefore, individuals with SUD might seek stronger (chemical) agents to cope. Interestingly, although the differences between the MPPUS score and the IAT score were not statistically significant when analyzed with tests for non-parametric distribution, the intensity of smartphone and internet use was visibly higher in the SUD group. It seems that among the SUD group, individuals used their smartphones to a great degree regardless of the level of neuroticism. It is possible that the use of smartphones among individuals with SUD may be associated with other motives, not just coping with negative emotions. This is consistent with the concept of addiction replacement, which claims that people who recover from one addiction are at increased risk of evolving to another form of addiction [38]. In other words, one may use their drug of choice to manage negative emotions, and there is a potential competition between other coping mechanisms, which may be either behavioral or chemical [39,40]. Specific motives for smartphone use among individuals with SUD could be an interesting area of future work. On the other hand, healthy individuals with higher levels of neuroticism, who do not use substances to cope with negative emotions, may use mobile phones for coping purposes and possibly find it effective in managing negative affect. Importantly, while there was support for moderation in the effect of SUD status on the association between neuroticism and PSU, this effect was not significant for the SUD group. Therefore, it can be speculated that persistent substance use among individuals with SUD reduced the otherwise significant association between neuroticism and PSU that was observed in the NS. Also, given the fact that individuals from the NS group were significantly younger than individuals with SUD, it would be interesting to examine their future mechanisms of coping with negative emotions.

### 4.2. Openness to New Experiences

Our findings indicate that higher openness to new experiences in the NS was significantly associated with more excessive use of mobile phone use. Among individuals with SUD, the significant effect was in the opposite direction. That is, high openness to new experiences was a protective factor associated with less intense mobile phone use.

The current study is only partially consistent with prior work. Previous research conducted on students demonstrated associations similar to the current study’s SUD group. Namely, openness to new experiences had a negative association with problematic mobile phone use [13,41]. In general, openness to new experiences refers to the tendency to be open-minded, imaginative, and curious [42]. The NS was comprised of Polish medical students that might use their smartphone as a tool for seeking novelty. Medical school is demanding and time-consuming; therefore, developing a social life via mobile tools often replace direct interactions, which is in fact normative for young individuals [43,44].

It is difficult to explain why our findings are inconsistent with prior scientific reports. One possible explanation is the timing of prior work with a majority of previous data gathered half a decade ago. It is known that generations differ in terms of their mobile phone use. In addition, to date, a comparative Polish sample to determine whether findings generalize is not available [45].

According to the literature, individuals with opioid use disorder tend to have average scores on the openness to new experiences domain [46]. In contrast, high levels of openness to new experiences are generally associated with the use of marijuana, hallucinogens, and stimulants [37,46]. People less open to new experiences tend to be conventional in their behavior and generally have a narrower range of interest [47]. It is worth mentioning that when comparing the NS and individuals with a SUD, those with a SUD were in general less open to new experiences than students. Importantly, as mentioned earlier, the SUD sample was significantly older (about 10 years on average) in comparison to the NS. Therefore, in this SUD sample, a “traditional” pattern (that is higher openness associated with less intense mobile phone/internet use), consistent with previous studies, takes place.

### 4.3. Extraversion, Agreeableness, Conscientiousness

Correlations between MPPUS-10 and other traits included in the Big Five questionnaire (i.e., extraversion, agreeableness, and conscientiousness) were consistent in terms of direction for both groups. Namely, there was a positive association between mobile phone use and extraversion, and a negative correlation with conscientiousness and agreeableness, which is in general consistent with prior scientific reports [16]. Also, personality characteristics among individuals with SUD in our study (high neuroticism, low openness, consciousness and agreeableness) was similar to those previously described for poly-drug users [20].

PSU is gaining increasing interest in recent years due to the irreplaceability and multifunctionality of smartphones. It is important to note that mobile phones also have many advantages and may have significant utility in helping to spread health-promoting behaviors and lifestyles. Paradoxically, they can also be used to implement screening and interventions aimed at managing substance-related problems [48]. Nevertheless, targeting individuals prone to excessive mobile phone use and implementing preventive programs is critical. Personality traits may be helpful in the screening process and the development of individualized treatments for specific groups. Our findings indicate that it may be useful in clinical practice to assess neuroticism and openness to new experiences among young adults. Addressing how to enhance new experiences in real, as opposed to virtual, life and how to effectively cope with negative emotional states without the use of smartphones could have utility. Clinical implications with regard to individuals with SUD remain to be further explored. However, our data support the assumption that behavioral and substance use addictions may influence one another and that a possible target for SUD treatments may be the implementation of less harmful, yet still stimulating/regulating behavioral activities, like physical exercises [49].

Prior work suggests that in the case of “non-chemical” rewards, physiological mechanisms (e.g., secretion of endogenous morphine-like substances) are critical, while most psychoactive substances directly affect neurotransmission, bypassing physiological pathways [50]. Thus, one can speculate that behavior-based addictions are less closely linked to increased neurotransmission, but more likely connected to personality traits. Our study suggests that the presence of SUD symptoms may affect this mechanism and change the associations between personality traits and addictive behaviors (i.e., mobile phone/internet use) observed in healthy individuals. Namely, high neuroticism and openness to new experiences increased the risk of PSU in healthy individuals, but not in individuals with SUD. Importantly, in our SUD sample, opioids were the drug of choice. Therefore, it can be speculated that in a SUD sample the needs associated with specific personality traits were addressed by the use of powerful exogenous opioids, rather than by activities enhancing endogenic morphine-like substances (like mobile phone use).

### 4.4. Limitations

The NS was comprised of medical students representing a homogeneous sample of individuals without problematic substance use that were a similar age and with good cognitive functioning. It can be assumed that most of them had, and used, a smartphone on a daily basis. In contrast, the SUD group was more heterogeneous and composed of poly-drug-users that mostly met criteria for an opioid use disorder. Level of education and comprehension of survey items were not verified. This represents an important limitation. Additionally, individuals with SUD were significantly older than the NS and the distribution of biological sex differed across the groups, yet age and biological sex were included as covariates across models. Nevertheless, future studies should compare groups that are more similar across biological sex. As observed in previous studies [51], women may generally score higher on all five personality factors. Moreover, men and women may use different strategies to cope with negative emotions, which may be meaningful as far as neuroticism is concerned [52]. Another limitation is a lack of information regarding whether all participants were using *smart* mobile devices or older generation products with limited functionality. Moreover, the literature does not show an unequivocal cut-off point for the MPPUS-10 scale, which may indicate addiction to mobile phone usage. The PSU operates on a continuum with a higher score representing more problematic use [27]. Therefore, no conclusions can be made in terms of diagnostic criteria of problematic mobile phone use in either group.

## 5. Conclusions

Results indicate that there were no significant differences in level of PSU between students and individuals with SUD, which might suggest different mechanisms underlying excessive mobile phone use. Both high neuroticism and openness to new experiences may increase the risk of PSU in healthy individuals, but not among individuals with SUD. Thus, the presence of SUD may influence how personality traits are associated with technological addictions. This observation should be replicated in further studies, especially among younger individuals with SUD.

## Figures and Tables

**Table 1:** Comparison of demographic, clinical characteristics, and personality traits between groups.

	NS Group *n* = 554	SUD Group *n* = 151	*p*-Value
Age	20 (19; 22)	31 (24; 36)	<0.0005
Biological sex [% male]	39.5	70.7	<0.0005
MMPUS-10	36 (26; 48)	36 (23; 59)	0.28
IAT	30 (26; 36)	30 (23; 47)	0.21
DUDIT	11 (11; 11)	42 (33; 46)	<0.0005
Neuroticism	25 (17; 32)	27 (23; 31)	<0.0005
Openness	30 (26; 35)	26 (22; 30)	<0.0005
Consciousness	33 (26; 38)	24 (21; 31)	<0.0005
Extraversion	27 (22; 32)	26 (21; 30)	0.068
Agreeableness	28 (24; 32)	25 (21; 29)	<0.0005

*Note.* MPPUS-10—Mobile Phone Problematic Use Scale; IAT—Internet Addiction Test; DUDIT—Drug Use Disorder Identification Test. NS—Normative Sample, SUD—Substance Use Disorder.

**Table 2:** Direct and interactive effects of personality traits and substance use disorder (SUD) status on mobile phone use.

	Model A: Openness on Mobile Phone Use	Model B: Neuroticism on Mobile Phone Use
***b*_1_**	*b*_1_ = 0.622	*b*_1_ = 0.666
	*p* < 0.001	*p* < 0.001
***b*_2_**	*b*_2_ = 17.741	*b*_2_ = 13.785
	*p* < 0.001	*p* < 0.001
***b*_3_**	*b*_3_ = −0.400	*b*_3_ = −0.268
	*p* < 0.001	*p* = 0.009
***b*_4_**	*b*_4_ = −0.830	*b*_4_ = −0.953
	*p* < 0.001	*p* < 0.001
***b*_5_**	*b*_5_ = 2881	*b*_5_ = 1817
	*p* = 0.023	*p* = 0.152

Values represent non-standardized coefficients Superscripts correspond to associations presented in Figure 4.

## Data Availability

The data presented in this study are available on request from the corresponding author.
